# Early prognostication of neurological outcome by heart rate variability in adult patients with out-of-hospital sudden cardiac arrest

**DOI:** 10.1186/s13054-019-2603-6

**Published:** 2019-10-17

**Authors:** Hiroshi Endoh, Natuo Kamimura, Hiroyuki Honda, Masakazu Nitta

**Affiliations:** 1 Department of Emergency & Critical Care Medicine, Niigata University Faculty of Medicine, 1-757 Asahimachi-dori, Chuo-ku, Niigata, 951-8520 Japan; 2 Advanced Emergency and Critical Care Center, Niigata University Medical & Dental Hospital, 1-754 Asahimachi-dori, Chuo-ku, Niigata, 951-8520 Japan

**Keywords:** Prognostication, Glasgow outcome scale, Heart rate variability, Targeted temperature management, Out-of-hospital sudden cardiac arrest

## Abstract

**Background:**

Most deaths of comatose survivors of out-of-hospital sudden cardiac arrest result from withdrawal of life-sustaining treatment (WLST) decisions based on poor neurological prognostication and the family’s intention. Thus, accurate prognostication is crucial to avoid premature WLST decisions. However, targeted temperature management (TTM) with sedation or neuromuscular blockade against shivering significantly affects early prognostication. In this study, we investigated whether heart rate variability (HRV) analysis could prognosticate poor neurological outcome in comatose patients undergoing hypothermic TTM.

**Methods:**

Between January 2015 and December 2017, adult patients with out-of-hospital sudden cardiac arrest, successfully resuscitated in the emergency department and admitted to the intensive care unit of the Niigata University in Japan, were prospectively included. All patients had an initial Glasgow Coma Scale motor score of 1 and received hypothermic TTM (at 34 °C). Twenty HRV-related variables (deceleration capacity; 4 time-, 3 geometric-, and 7 frequency-domain; and 5 complexity variables) were computed based on RR intervals between 0:00 and 8:00 am within 24 h after return of spontaneous circulation (ROSC). Based on Glasgow Outcome Scale (GOS) at 2 weeks after ROSC, patients were divided into good outcome (GOS 1–2) and poor outcome (GOS 3–5) groups.

**Results:**

Seventy-six patients were recruited and allocated to the good (*n* = 22) or poor (*n* = 54) outcome groups. Of the 20 HRV-related variables, ln very-low frequency (ln VLF) power, detrended fluctuation analysis (DFA) (α1), and multiscale entropy (MSE) index significantly differed between the groups (*p* = 0.001), with a statistically significant odds ratio (OR) by univariate logistic regression analysis (*p* = 0.001). Multivariate logistic regression analysis of the 3 variables identified ln VLF power and DFA (α1) as significant predictors for poor outcome (OR = 0.436, *p* = 0.006 and OR = 0.709, *p* = 0.024, respectively). The area under the receiver operating characteristic curve for ln VLF power and DFA (α1) in predicting poor outcome was 0.84 and 0.82, respectively. In addition, the minimum value of ln VLF power or DFA (α1) for the good outcome group predicted poor outcome with sensitivity = 61% and specificity = 100%.

**Conclusions:**

The present data indicate that HRV analysis could be useful for prognostication for comatose patients during hypothermic TTM.

## Background

Despite progress in practices of cardiopulmonary resuscitation and post-cardiac arrest care, most survivors of out-of-hospital sudden cardiac arrest remain comatose due to severe hypoxic-ischemic brain injury [1]. However, most deaths in these patients result from withdrawal of life-sustaining treatment (WLST) decisions based on poor neurological prognostication and the family’s intention [2, 3]. Thus, accurate prognostication of poor neurological outcome is crucial not only to avoid premature WLST decisions, but also to avoid unnecessary examinations or expensive treatments and lengthy anxious waiting periods for families of patients who will have a poor outcome.

Recent cardiopulmonary resuscitation guidelines strongly recommend targeted temperature management (TTM) for comatose survivors after return of spontaneous circulation (ROSC) [4, 5]. TTM with sedation or a neuromuscular blocking agent for control of shivering significantly affects early and accurate prognostication [6, 7]. The diagnostic accuracy of a robust prognosticator for poor outcome is recommended to have a specificity of > 95% (negative predicting value < 5%) in clinical settings [6–8].

Traditionally, heart rate variability (HRV) provides a convenient and noninvasive method to assess the balance between sympathetic and parasympathetic activities of the autonomic nervous system [9]. Numerous studies have shown that time- or frequency-domain variables of HRV can reflect clinical severity and prognosis in intensive care unit (ICU) patients [10, 11]. Recently, complexity variables based on the nonlinear fractal dynamics of human HRV have been shown to provide additional prognostic information and complement traditional time- and frequency-domain variables [12, 13].

Several prior studies have indicated that HRV-related variables may predict the outcome of comatose patients after ROSC. Huikuri et al. reported that ROSC patients had a lower standard deviation of all RR intervals (SDNN) or high-frequency power per 24 h compared with non-cardiac arrest patients [14]. Dougherty and Burr reported that the SDNN and low-frequency power per 24 h were significantly related to 1-year mortality [15]. Chen et al. showed that normalized low-frequency power of a 10-min RR interval was a significant predictor of 24-h mortality [16]. However, the post ROSC patients in these studies were not treated with hypothermic TTM.

In the present study, we investigated HRV-related prognosticators within 24 h after ROSC in patients with an initial Glasgow Coma Scale (GCS) motor score of 1 undergoing hypothermic TTM.

## Methods

This prospective, observational, single-center study was approved by the local ethical committee of the Medical Faculty of Niigata University.

### Participants

Between January 1, 2015, and December 31, 2017, adult patients with out-of-hospital sudden cardiac arrest who were transferred to the emergency department at Niigata University Hospital and successfully resuscitated were consecutively enrolled. All resuscitated patients were admitted to the Niigata University Hospital ICU.

Patients were eligible for participation if they met the following criteria: more than 16 years old, GCS motor scale of 1 at first evaluation after ROSC, and sinus rhythm. Exclusion criteria were as follows: traumatic cardiac arrest, cardiac arrest due to cerebral origins, normothermic TTM (36 °C), new onset of atrial fibrillation or atrial flutter rhythm, and hemodynamically unstable patients (severe hypotension).

### Study protocol

All patients were mechanically ventilated to maintain normocapnia (PaCO_2_ 35–45 mmHg) under sedation with continuous intravenous infusion of midazolam (0.1–0.2 mg kg^−1^ h^−1^) with fentanyl (0.1–0.2 μg kg^−1^ h^−1^). Rocuronium bromide was continuously infused to mitigate uncontrollable shivering (500–750 μg kg^−1^ h^−1^).

TTM targeting 34 °C of bladder temperature for 24 h was introduced and maintained with an intravascular cooling device (Thermogard XP®, Asahikasei Zoll medical, Japan) or a body surface cooling device (Arctic Sun 2000®, IMI, Japan). During hypothermia, a mean arterial pressure (MAP) > 60 mmHg was maintained with fluid resuscitation and/or continuous infusion of noradrenaline. All patients were rewarmed at a rate of 0.25 °C h^−1^. Patients who could not maintain MAP > 60 mmHg with these methods were excluded from the study. The treating ICU physicians were blinded to the following HRV-related metrics during study period.

### Assessment of decelerating capacity; time-, frequency-, and geometrical-domain; and complexity variables of HRV

Electrocardiograms (ECG) were continuously monitored with a bedside monitor (IntelliVue MP70®, Philips, Japan), and the ECG wave data were captured at a sampling frequency of 250 Hz with 14-bit resolution and automatically stored in the dedicated server. RR intervals between 0:00 am and 8:00 am within the first 24 h post-ROSC were identified by wqrs algorithm [17] and stored as a comma-separated value file (CSV) after 5 points moving averaging. The 8-h recording was started at midnight because this timeframe had fewer external stimuli such as physiological examinations or family visits.

Sinus rhythm was considered only when RR intervals were between 300 and 2000 ms and differed ≤ 20% from the average of five preceding sinus rhythm RR intervals, and consecutive RR interval differences were ≤ 200 ms [18]. Any RR intervals not based on the above sinus rhythm criteria were replaced with the average value of the five preceding sinus rhythm RR intervals. When the replacement number divided by the entire RR intervals (replacement ratio) was > 20%, the patient was excluded from this study.

Decelerating capacity (DC) was computed using the software program calc-prsa (version 1.3.0) that was developed based on the phase-rectified signal averaging technique [19].

The method used for time-, frequency-, and geometric-domain HRV analysis has been described elsewhere and adhered to the standards developed by the Task Force of the European Society of Cardiology and the North American Society of Pacing and Electrophysiology [20].

For the time-domain variables, the average of all RR intervals (AVNN), the SDNN, square root of the mean of the squares of differences between adjacent RR intervals (rMSSD), and the percentage of differences between adjacent RR intervals > 50 ms (pNN50) were computed.

For the frequency-domain variables, a Lomb-Scargle periodogram was plotted to measure the spectral power of the ultra-low-frequency range (ULF, 0–0.003 Hz), the very-low-frequency range (VLF, 0.003–0.04 Hz), the low-frequency range (LF, 0.04–0.15 Hz), the high-frequency range (HF, 0.15–0.4 Hz), the total power (TP, 0–0.4 Hz), the ratio of low- to high-frequency power (LF/HF), and the slope of the linear interpolation between 10^−4^ and 10^−2^ Hz of the spectrum in a log-log scale (power-law slope, exponent β). All measured powers were expressed as natural logarithm (ln).

For the geometric-domain variables, the total number of all RR intervals was divided by the height of the histogram of all RR intervals measured on a discrete scale with bins of 7.185 ms (triangular index), and RR interval was plotted as a function of the previous one (Poincaré plot). SD1 and SD2 are the two dispersions (standard deviations [SD]) of projections of the Poincaré plot on the line of identity (*y* = *x*) and on the line perpendicular to the line of identity (*y* = − *x*), respectively [21].

For the complexity variables, approximate entropy (ApEn) and sample entropy (SampEn) were computed with a parameter of *m* = 2 and similarity criterion = 20% of SD [22]. Multiscale entropy (MSE) index was defined as the sum of the sample entropy at a scale factor of 1–20 [23]. Detrended fluctuation analysis (DFA) was measured to quantify fractal scaling properties of the RR interval [24]. The scaling properties were defined separately for short-term (4 ≤ *n* ≤ 16 beats, *α*_1_) and long-term (*n* > 16 beats, *α*_2_) RR intervals.

DC, rMSSD, pNN50, ln LF power, ln HF power, and LF/HF were computed from the segment of 512 RR intervals, and the averaged values of the entire RR intervals were calculated. AVNN, SDNN, triangular index, ln total power, ln ULF power, ln VLF power, ApEn, SampEn, and MSE index were computed for the entire RR intervals. SD1, SD2, DFA (α_1_), and DFA (α_2_) were computed from the segment of 1000 RR intervals, and the averaged values of the entire RR intervals were calculated.

All variables except DC were computed with programs downloaded from PhysioNet (https://physionet.org /physiotools/matlab/wfdb/wfdb-app-matlab/).

### Study endpoint

The primary endpoint was Glasgow Outcome Scale (GOS) on the 14th day after ROSC. The good outcome group included patients with a good recovery (GOS 1) or moderate disability (GOS 2). The poor outcome group included patients with severe disability (GOS 3), a persistent vegetative state (GOS 4), or death (GOS 5). The GOS level was prospectively assessed by ICU physicians until death or hospital discharge.

### Statistical analyses

Categorical variables were presented as numbers or percentage and compared using the chi-squared test or Fisher’s exact test. Continuous variables were presented as median (interquartile range) and compared using the Mann-Whitney *U* test. Univariate logistic regression analysis for poor outcome was performed on each HRV-related variable. Variables were included in the multivariate logistic regression analysis if *p* = 0.001 (both Mann-Whitney *U* test and univariate logistic regression analysis). Results were presented as odds ratios (OR) and 95% confidence intervals (CI).

The receiver operating characteristic (ROC) curve was plotted, and the area under the curve (AUC) was calculated to evaluate the predictive performance of HRV-related variables for poor outcome. Youden index was used to calculate the optimal cut off value. Sensitivity and specificity were determined for the selected cutoff value. In addition, sensitivity, specificity, positive predictive value (PPV), negative predictive value (NPV), and false-positive ratio (FPR) for poor outcome were calculated by dichotomy of the minimum values of the patients with good outcome.

Two-sided *p* values < 0.05 were considered statistically significant. All analyses were performed using STATA/SE package version 15.0 (StataCorp, College Station, TX, USA).

## Results

### Patient characteristics

**Fig. 1:**
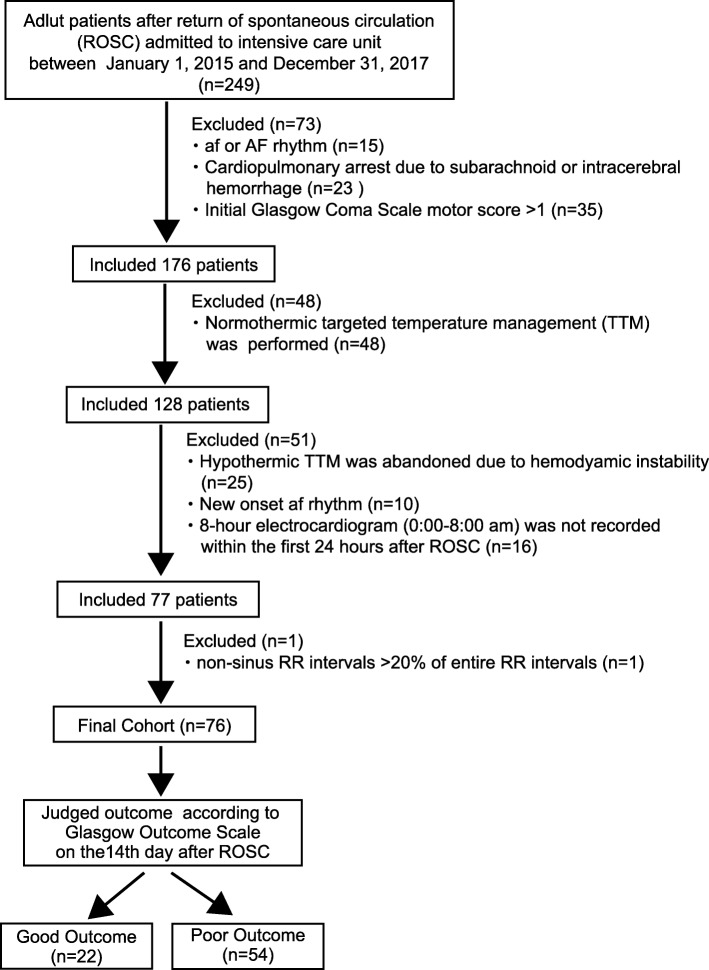
Patient flow chart

After the exclusion of patients, a total of 77 patients were recruited; however, one patient with replacement rate > 20% was excluded. Thus, 76 patients were enrolled and divided by GOS on the 14th day after ROSC into good outcome (*n* = 22) or poor outcome (*n* = 54) groups (Fig. 1).

The median replacement rate of good or poor outcome groups was 0.13% or 0.38%, respectively, with no statistically significant difference (*p* = 0.642).

Patient characteristics are shown in Table 1. There was no significant difference in age, gender, Acute Physiology and Chronic Health Evaluation (APACHE) II score, and Sequential Organ Failure Assessment (SOFA) Score (day 1) between good and poor outcome groups.

**Table 1:** Comparison of patient characteristics and HRV-related variables between good and poor outcomes

Patient characteristics /HRV-related variable	Total (*n* = 76)	Good outcome (*n* = 22)	Poor outcome (*n* = 54)	*p*
Age (years)	61 (46–76)	58 (49–72)	61 (45–77)	0.748
Gender (F/M) (*n*)	26/50	7/15	19/35	0.779
APACHE II score	28 (26–31)	27 (26–29)	28 (27–32)	0.185
SOFA score, day 1	11 (8–13)	10 (7–11)	11 (8–14)	0.153
DC	1.83 (0.92–3.34)	2.69 (1.23–5.32)	1.64 (0.78–2.77)	0.037
AVNN (ms)	699 (605–827)	804 (698–842)	677 (574–788)	0.009
SDNN (ms)	40.9 (25.3–57.8)	47.6 (34.7–71.5)	35.5 (23.8–55.4)	0.090
rMSSD (ms)	10.4 (4.9–19.8)	15.3 (6.4–24.4)	9.1 (4.2–17.0)	0.111
pNN50(%)	0.88 (0.16–3.76)	1.17 (0.16–3.76)	0.77 (0.16–3.47)	0.518
Triangular index	7.3 (5.1–11.1)	9.8 (6.7–15.3)	6.6 (4.9–10.3)	0.018
Poincaré plot, SD1 (ms)	7.5 (3.5–14.8)	11.5 (4.5–19.7)	7.2 (3.1–12.9)	0.088
Poincaré plot, SD2 (ms)	17.4 (10.0–29.9)	31.0 (21.1–43.0)	13.3 (7.4–22.6)	0.001
ln total power (ms^2^)	8.06 (7.15–8.80)	8.48 (7.77–9.46)	7.84 (7.03–8.70)	0.062
ln ULF power (ms^2^)	7.83 (6.90–8.63)	8.06 (7.39–8.81)	7.72 (6.80–8.59)	0.140
ln VLF power (ms^2^)	4.83 (3.73–5.98)	6.06 (5.49–7.12)	4.41 (3.14–5.22)	0.001
ln LF power (ms^2^)	3.65 (1.72–4.59)	4.38 (3.54–5.30)	3.10 (1.36–4.33)	0.002
ln HF power (ms^2^)	3.53 (2.39–4.72)	3.96 (2.71–5.08)	3.11 (1.99–4.67)	0.195
LF/HF	0.98 (0.52–2.04)	2.23 (1.10–3.72)	0.73 (0.40–1.42)	0.001
Power law (*β*)	− 1.45 (− 1.74 to − 1.20)	− 1.36 (− 1.48 to − 1.05)	− 1.56 (− 1.80 to − 1.31)	0.015
DFA (*α*_1_)	0.79 (0.64–0.98)	0.98 (0.87–1.22)	0.71 (0.60–0.86)	0.001
DFA (*α*_2_)	0.92 (0.77–1.12)	1.10 (0.88–1.29)	0.88 (0.71–1.01)	0.003
ApEn	0.55 (0.27–0.84)	0.59 (0.30–0.95)	0.51 (0.26–0.81)	0.336
SampEn	0.37 (0.17–0.60)	0.38 (0.23–0.79)	0.36 (0.16–0.55)	0.348
MSE index	10.8 (3.5–19.4)	19.0 (14.8–22.6)	8.3 (2.9–13.6)	0.001

All values are expressed as *n* or median (interquartile range)

*Abbreviations*: *AVNN* average of all RR intervals, *APACHE* Acute Physiology and Chronic Health Evaluation, *ApEn* approximate entropy, *DC* decelerating capacity, *DFA* detrended fluctuation analysis, *F* female, *HF* high frequency, *HRV* heart rate variability, *LF* low frequency, *LF/HF* ratio of low- to high-frequency power, *ln* natural logarithm, *M* male, *MSE* multiscale entropy, *Power law* slope of the regression of power spectrum in log-log scale, *pNN50* % of successive RR intervals differing > 50 ms, *rMSSD* square root of the mean of the squares of differences between adjacent RR intervals, *SampEn* sample entropy, *SDNN* standard deviation of all RR intervals, *SD1 and SD2* standard deviations of short and long axis of Poincaré plot, *SOFA* Sequential Organ Failure Assessment, *Triangular index* total number of all RR intervals divided by the height of the histogram of all RR intervals, *ULF* ultra-low frequency, *VLF* very-low frequency

### Comparisons of HRV-related variables between poor and good outcomes

Table 1 also shows comparisons of 20 HRV-related variables. There was a significant difference in DC (*p* = 0.037), AVNN (*p* = 0.009), triangular index (*p* = 0.018), SD2 (*p* = 0.001), ln VLF power (*p* = 0.001), ln LF power (*p* = 0.002), LF/HF (*p* = 0.001), power law (*β*) (*p* = 0.015), DFA (α_1_) (*p* = 0.001), DFA (*α*_2_) (*p* = 0.003), and MSE index (*p* = 0.001) between good and poor outcome groups. The curves of the MSE of poor and good outcome groups are shown in Additional file 1: Figure S1.

Table 2 shows OR and 95% CI of 20 HRV-related variables for poor outcome by univariate logistic regression analysis. Significant univariate variables for poor outcome were DC (*p* = 0.033), AVNN (*p* = 0.012), ln total power (*p* = 0.032), ln VLF power (*p* = 0.001), ln LF power (*p* = 0.003), LF/HF (*p* = 0.002), DFA (α_1_) (*p* = 0.001), DFA (α_2_) (*p* = 0.003), and MSE index (*p* = 0.001).

**Table 2:** Univariate and multivariate logistic regression analyses of HRV-related variables for prediction of poor outcome

HRV-related variable	Odds ratio (95% CI)	*z*	*p*
Univariate logistic regression analysis			
DC	0.819 (0.681–0.984)	− 2.13	0.033
AVNN (ms)	0.995 (0.992–0.999)	− 2.52	0.012
SDNN (ms)	0.990 (0.977–1.003)	− 1.53	0.127
rMSSD (ms)	0.965 (0.929–1.003)	− 1.81	0.070
pNN50(%)	0.985 (0.924–1.051)	− 0.45	0.654
Triangular index	0.915 (0.834–1.004)	− 1.89	0.059
Poincaré plot, SD1 (ms)	0.982 (0.951–1.015)	− 1.06	0.288
Poincaré plot, SD2 (ms)	0.985 (0.968–1.001)	− 1.79	0.073
ln total power (ms^2^)	0.626 (0.409–0.960)	− 2.15	0.032
ln ULF power (ms^2^)	0.700 (0.469–1.043)	− 1.75	0.080
ln VLF power (ms^2^)	0.373 (0.224–0.620)	− 3.80	0.001
ln LF power (ms^2^)	0.598 (0.424–0.845)	− 2.92	0.003
ln HF power (ms^2^)	0.802 (0.593–1.084)	− 1.44	0.151
LF/HF	0.472 (0.304–0.733)	− 3.35	0.002
Power law (*β*)	0.278 (0.074–1.038)	− 1.90	0.057
DFA (*α*_1_) (per 0.1 U)	0.635 (0.501–0.805)	− 3.76	0.001
DFA (*α*_2_) (per 0.1 U)	0.702 (0.556–0.886)	− 2.98	0.003
ApEn (per 0.1 U)	0.930 (0.820–1.055)	− 1.13	0.260
SampEn (per 0.1 U)	0.911 (0.787–1.054)	− 1.25	0.211
MSE index	0.873 (0.811–0.941)	− 3.56	0.001
Multivariate logistic regression analysis			
Ln VLF power	0.436 (0.242–0.784)	− 2.77	0.006
DFA (*α*_1_) (per 0.1 U)	0.709 (0.523–0.956)	− 2.26	0.024
MSE index	0.983 (0.888–1.088)	− 0.33	0.739

*Abbreviations*: *AVNN* average of all RR intervals, *ApEn* approximate entropy, *CI* confidence interval, *DC* decelerating capacity, *DFA* detrended fluctuation analysis, *HF* high frequency, *HRV* heart rate variability, *LF* low frequency, *LF/HF* ratio of low to high frequency power, *ln* natural logarithm, *MSE* multiscale entropy, *Power law* slope of the regression of power spectrum in log-log scale, *pNN50* % of successive RR intervals differing > 50 ms, *rMSSD* square root of the mean of the squares of differences between adjacent RR intervals, *SampEn* sample entropy, *SDNN* standard deviation of all RR intervals, *SD1and SD2* standard deviations of short and long axis of Poincaré plot, *Triangular index* total number of all RR intervals divided by the height of the histogram of all RR intervals, *ULF* ultra-low frequency, *VLF* very-low frequency

Table 2 also shows OR and 95% CI of variables by multivariate logistic regression analysis of the 3 variables that were statistically significant by both Mann-Whitney *U* test and univariate logistic regression analysis (*p* = 0.001). ln VLF power and DFA (α_1_) were significant predictors for poor outcome (OR = 0.436, *p* = 0.006 and OR = 0.709, *p* = 0.024, respectively).

**Fig. 2:**
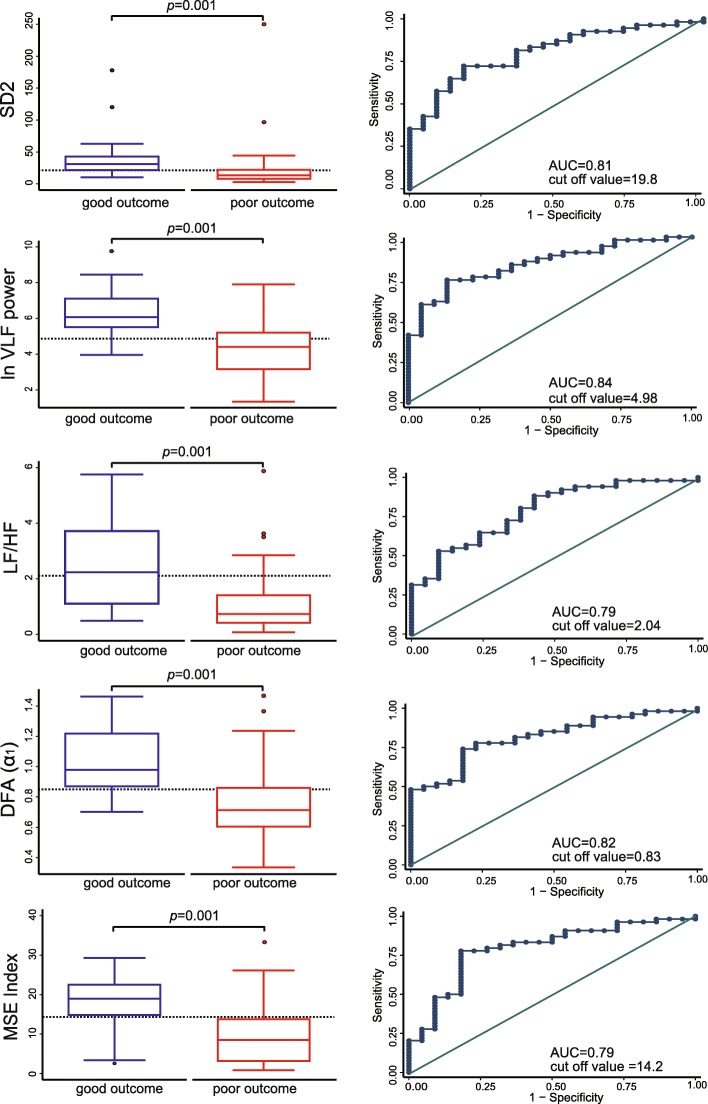
Box-and-whisker plot, ROC curve, AUC, and cutoff value for the 5 HRV-related variables that were statistically significant by Mann-Whitney *U* test (*p* = 0.001). The horizontal dotted lines in box-and-whisker plot indicate cutoff values

Figure 2 shows a box-and-whisker plot, ROC curve, AUC, and optimal cutoff value for the 5 HRV-related variables that were statistically significant by Mann-Whitney *U* test (*p* = 0.001). The AUC for ln VLF power and DFA (α1) were 0.84 (95%CI = 0.75–0.93) and 0.82 (95%CI = 0.72–0.91), respectively. The combination of both variables yielded a higher predictive performance (AUC = 0.88, 95%CI = 0.80–0.95). The AUC, cutoff value, sensitivity, and specificity of each HRV-related variable are shown in Additional file 1: Table S1.

**Fig. 3:**
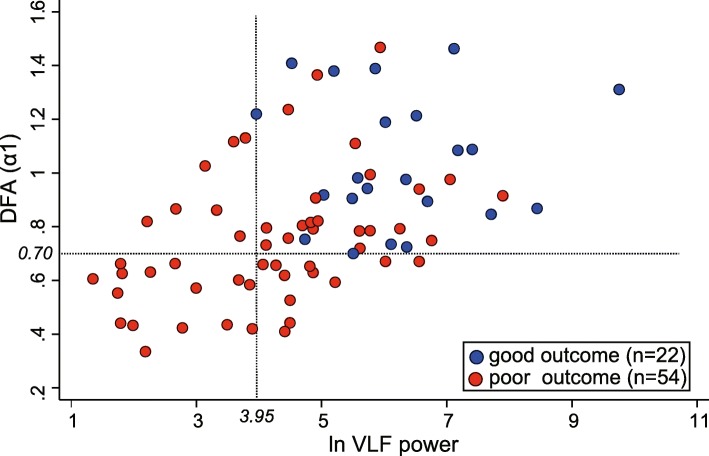
Scatter plot of patients with good or poor outcome (*n* = 76) with corresponding values for ln VLF (*x*-axis) and DFA (*α*_1_) (*y*-axis). Dashed lines indicate the minimal value of patients with good outcome

Figure 3 shows scatter plots of good or poor outcome patients with corresponding values for ln VLF power (*x*-axis) and DFA (*α*_1_) (*y*-axis). The dotted lines indicate the minimal value of the patients with good outcome. ln VLF power < 3.95 or DFA (α_1_) < 0.70 predicted poor outcome with sensitivity = 61%, specificity = 100%, PPV = 100%, NPV = 51%, and FPR = 0%.

## Discussion

We conducted this prospective and exhaustive study to identify early prognosticators among 20 HRV-related variables in post-ROSC comatose patients undergoing hypothermic TTM. Consequently, ln VLF power and DFA (α_1_) were significant predictors of poor outcome (OR = 0.463, *p* = 0.006, and OR = 0.709, *p* = 0.024, respectively), with a predictive ability (AUC = 0.84 and 0.82, respectively). In addition, the minimal value of ln VLF power or DFA (*α*_1_) for the patients with good outcome could predict poor outcome with sensitivity = 61% and specificity = 100% (Fig. 3), satisfying the requirements for a robust predictor of poor outcome [6–8].

The physiological bases for ULF and VLF power are less clear than LF and HF power. However, ln VLF power has been suggested to be a strong risk predictor in patients with reduced left ventricular ejection fraction after acute myocardial infarction [25], post aortic surgery [26], or multiple organ dysfunction (MODF) [27]. DFA is a fractal correlation characterized as free from external interference and requiring non-stationarity. Perkiömäki et al. concluded that a short-term fractal scaling exponent (α_1_) predicted fatal cardiovascular events in various populations and might provide more prognostic information than traditional HRV indexes [28]. For example, in a prospective, multicenter study evaluating HRV as a predictor of death after acute myocardial infarction, reduced *α*_1_ (< 0.75) was the most powerful predictor of mortality [29].

Mechanical ventilation or sedation significantly suppresses HRV. Kasaoka et al. showed that LF and HF power and LF/HF per 5 min were significantly higher when ICU patients were breathing spontaneously after extubation [30]. Bradley et al. showed that ICU patients with a low or medium degree of MODF had a greater increase in HRV during sedation interruption, compared with a high degree of MODF [31]. Based on these findings, HRV may have been depressed in our study. However, the depression might be not so severe that differences between outcomes would have been undetectable.

Although hypothermia causes bradycardia physiologically, time-domain variables or frequency-domain variable of HRV during moderate hypothermia was enhanced in patients with poikilothermia [32] or healthy volunteers [33]. Tianien et al. reported that all HRV values based on 24-h RR intervals are higher in post-ROSC comatose patients treated with moderate hypothermia (33 °C) than those treated with normothermia (< 38 °C) [34]. In infants with hypoxic ischemic encephalopathy, increased HF power and AVNN during hypothermia were reported in infants with moderate brain injury [35] or favorable outcome [36] Thus, enhanced HRV produced by moderate hypothermia may counter-act the suppressive effects of mechanical ventilation with sedation in the present study.

### Limitations

Several limitations might affect the present findings. First, this study was conducted at a single institution with a small sample size. Thus, the cutoff values for HRV herein need to be validated in other studies with a larger cohort. Second, 20% of patients treated with hypothermic TTM were excluded due to severe hypotension, which may limit the applicability of the data of this study. Third, the 8-h recording time for RR intervals does not strictly adhere to the standard recommendation for HRV measures [20]. Consequently, long-term (24 h) HRV variables of SDNN, ULF power, and triangular index may become unreliable. Finally, not all patients had reached the targeted bladder temperature of 34 °C at the beginning of recording of RR intervals because the recording was scheduled to start at midnight within 24 h after ROSC.

## Conclusions

The present data indicate that HRV analysis could be useful for early prognostication for comatose patients during hypothermic TTM within 24 h after ROSC. The value of HRV as a prognosticator of poor outcome should be confirmed in a larger study.

## Supplementary information

**Additional file 1.** **Figure S1.** MSE curves for poor and good outcome groups. (EPS 1546 kb)

**Additional file 2.** Table S1. AUC, cut off value, sensitivity, and specificity of HRV- related variables for poor outcome. (DOCX 18 kb)

## Data Availability

Please contact the corresponding author for data requests.

## Abbreviations

APACHE: Acute Physiology and Chronic Health Evaluation
ApEn: Approximate entropy
AUC: Area under the curve
AVNN: Average of all RR intervals
CI: Confidential interval
DC: Decelerating capacity
DFA: Detrended fluctuation analysis
ECG: Electrocardiogram
FPR: False-positive ratio
GCS: Glasgow Coma Scale
GOS: Glasgow Outcome Scale
HF: High frequency
HRV: Heart rate variability
ICU: Intensive care unit
LF: Low frequency
LF/HF: Ratio of low- to high-frequency power
ln: Natural logarithm
MAP: Mean arterial pressure
MODF: Multiple organ dysfunction
MSE: Multiscale entropy
NPV: Negative predictive value
OR: Odds ratio
Power law: Slope of the regression of power spectrum in log-log scale
pNN50: Percentage of differences between adjacent RR intervals > 50 ms
PPV: Positive predictive value
rMSSD: Square root of the mean of the squares of differences between adjacent RR intervals
ROC: Receiver operating characteristic
ROSC: Return of spontaneous circulation
SampEn: Sample entropy
SD: Standard deviation
SDNN: Standard deviation of all RR intervals
SD1 and SD1: Standard deviations of short and long axis of Poincaré plot
SOFA: Sequential Organ Failure Assessment
TP: Total power
Triangular index: Total number of all RR intervals divided by the height of the histogram of all RR intervals
TTM: Targeted temperature management
ULF: Ultra-low frequency
VLF: Very-low frequency
