# Supplementary material for: Early prognostication of neurological outcome by heart rate variability in adult patients with out-of-hospital sudden cardiac arrest
Source: Crit Care. 2019 Oct 17;23:323. doi: 10.1186/s13054-019-2603-6 (PMC6798365; doi:10.1186/s13054-019-2603-6)
Supplement: Supplementary file 2 — Additional file 2. Table S1. AUC, cut off value, sensitivity, and specificity of HRV- related variables for poor outcome. (DOCX 18 kb) [file 13054_2019_2603_MOESM2_ESM.docx]

**Table S1. AUC, cut off value, sensitivity, and specificity of HRV- related variables for poor outcome**

| HRV-related Variables | AUC (95%CI) | Cut off value | Sensitivity% | Specificity% |
| --- | --- | --- | --- | --- |
| DC | 0.66 (0.51-0.80) | 3.2 | 48 | 82 |
| AVNN (ms) | 0.70 (0.58-0.83) | 661 | 91 | 48 |
| SDNN (ms) | 0.62 (0.49-0.76) | 34.5 | 77 | 50 |
| rMSSD (ms) | 0.62 (0.47-0.77) | 15.1 | 73 | 62 |
| pNN50(%) | 0.55 (0.39-0.70) | 0.82 | 67 | 52 |
| Triangular index | 0.67(0.54-0.80) | 6.31 | 86 | 46 |
| Poincaré plot, SD1 (ms) | 0.63 (0.48-0.77) | 10.9 | 55 | 72 |
| Poincaré plot, SD2 (ms) | 0.81 (0.71-0.91) | 19.8 | 82 | 72 |
| ln total power (ms^2^) | 0.64 (0.50-0.77) | 7.76 | 77 | 48 |
| ln ULF power (ms^2^) | 0.61 (0.47-0.74) | 6.55 | 100 | 24 |
| ln VLF power (ms^2^) | 0.84 (0.75-0.93) | 4.98 | 86 | 74 |
| ln LF power (ms^2^) | 0.73 (0.61-0.85) | 3.34 | 81 | 55 |
| ln HF power (ms^2^) | 0.60 (0.45-0.74) | 5.51 | 24 | 92 |
| LF/HF | 0.79 (0.67-0.90) | 2.04 | 57 | 88 |
| Power law (β) | 0.68 (0.55-0.81) | -1.51 | 82 | 56 |
| DFA (α_1_) | 0.82 (0.72-0.91) | 0.83 | 82 | 74 |
| DFA (α_2_) | 0.72 (0.60-0.85) | 1.07 | 59 | 89 |
| ApEn | 0.57 (0.42-0.72) | 0.82 | 41 | 78 |
| SampEn | 0.57 (0.42-0.72) | 0.30 | 73 | 43 |
| MSE index | 0.79 (0.68-0.91) | 14.2 | 82 | 78 |

*Abbreviations*: AVNN = average of all RR intervals, ApEn = approximate entropy, AUC = area under curve, CI = confidence interval, DC = decelerating capacity, DFA = detrended fluctuation analysis, HF = high frequency, LF = low frequency, LF/HF = ratio of low to high frequency power, ln = natural logarithm, MSE = multiscale entropy, Power law = slope of the regression of power spectrum in log-log scale, pNN50 = % of successive RR intervals differing >50 ms, ROC=receiver operating characteristic, rMSSD =square root of the mean of the squares of differences between adjacent RR intervals, SampEn = sample entropy, SDNN = standard deviation of all RR intervals, SD1and SD2 = standard deviations of short and long axis of Poincaré plot, Triangular index = total number of all RR intervals divided by the height of the histogram of all RR intervals , ULF = ultra-low frequency, VLF = very-low frequency.
